# Recent Advances in EPAC-Targeted Therapies: A Biophysical Perspective

**DOI:** 10.3390/cells8111462

**Published:** 2019-11-19

**Authors:** Alveena Ahmed, Stephen Boulton, Hongzhao Shao, Madoka Akimoto, Amarnath Natarajan, Xiaodong Cheng, Giuseppe Melacini

**Affiliations:** 1 Department of Biochemistry and Biomedical Sciences, McMaster University, Hamilton, ON L8S 4L8, Canada; ahmeda64@mcmaster.ca (A.A.); boultos777@gmail.com (S.B.); 2 Department of Chemistry and Chemical Biology, McMaster University, Hamilton, ON L8S 4L8, Canada; shaoh1@mcmaster.ca (H.S.); akimotma@gmail.com (M.A.); 3 Eppley Institute for Research in Cancer and Allied Diseases, University of Nebraska Medical Center, Omaha, NE 68198, USA; anatarajan@unmc.edu; 4 Department of Integrative Biology & Pharmacology, University of Texas Health Science Center at Houston, Houston, TX 77030, USA; Xiaodong.Cheng@uth.tmc.edu; 5 Texas Therapeutics Institute, Institute of Molecular Medicine, University of Texas Health Science Center at Houston, Houston, TX 77030, USA

**Keywords:** EPAC, aggregation-based inhibition, allostery, cAMP, dynamics, drug design, NMR, protein-ligand binding, screening, signaling

## Abstract

The universal second messenger cAMP regulates diverse intracellular processes by interacting with ubiquitously expressed proteins, such as Protein Kinase A (PKA) and the Exchange Protein directly Activated by cAMP (EPAC). EPAC is implicated in multiple pathologies, thus several EPAC-specific inhibitors have been identified in recent years. However, the mechanisms and molecular interactions underlying the EPAC inhibition elicited by such compounds are still poorly understood. Additionally, being hydrophobic low molecular weight species, EPAC-specific inhibitors are prone to forming colloidal aggregates, which result in non-specific aggregation-based inhibition (ABI) in aqueous systems. Here, we review from a biophysical perspective the molecular basis of the specific and non-specific interactions of two EPAC antagonists—CE3F4R, a non-competitive inhibitor, and ESI-09, a competitive inhibitor of EPAC. Additionally, we discuss the value of common ABI attenuators (e.g., TX and HSA) to reduce false positives at the expense of introducing false negatives when screening aggregation-prone compounds. We hope this review provides the EPAC community effective criteria to evaluate similar compounds, aiding in the optimization of existing drug leads, and informing the development of the next generation of EPAC-specific inhibitors.

## 1. Introduction

Cyclic adenosine monophosphate (cAMP) is a universal second messenger that, upon interaction with an evolutionarily conserved cAMP binding domain (CBD) [1], tightly regulates a diverse array of physiological processes: muscle contraction [2], metabolism [3,4], calcium homeostasis [5], cell apoptosis [6], immune regulation [7], cell adhesion [8], secretion [9] and the regulation of gene expression [10]. Mediation of cAMP signaling was initially attributed mainly to the activation of two primary protein families: cAMP-dependent protein kinase A (PKA) [10,11] and cyclic nucleotide-activated channels, e.g., hyperpolarization activated cyclic nucleotide gated channels (HCN) and cyclic nucleotide gated channels (CNG) [12,13]. Additional cAMP-binding proteins have since been identified, e.g., the Popeye domain containing (Popdc) proteins and cyclic nucleotide receptor involved in sperm function (CRIS), underscoring the complexity of cAMP-related signaling [14,15]. Importantly, in 1998, a new cAMP receptor was discovered after observing cAMP-dependent activation for the small GTPase Rap1, which was independent of PKA [16]. The new cAMP receptor, now known as the exchange protein directly activated by cAMP (EPAC), was then identified through a database screen for putative cAMP-binding domains [16]. There are two isoforms of EPAC (EPAC1 and EPAC2) that serve as guanine exchange factors for Ras-like GTPases, primarily Rap1 and Rap2 [16,17]. EPAC1 is more broadly expressed, e.g., in the circulatory, excretory, reproductive and digestive systems, whereas EPAC2 is more abundant in the central nervous system (CNS), pancreas and adrenal gland [16,17].

EPAC regulates several critical cAMP-related signaling pathways, including cardiac calcium homeostasis, vascular remodeling, tissue homeostasis, regulation of smooth muscle contraction, insulin secretion from pancreatic β cells, integrin-dependent cell adhesion, neuronal excitability and memory consolidation in the hippocampus [8,18,19,20,21,22,23]. Thus, EPAC inhibition has been recognized as a promising therapeutic route for a wide spectrum of diseases, including pancreatic cancer [24], breast cancer [25], cardiac hypertrophy [26], vascular inflammation [27], Alzheimer’s disease [28], as well as infections, e.g., Coronavirus’ [29] and malaria [30]. For further details about the physiological role of EPAC and subsequent disease implications, we refer to excellent reviews, which have been published in recent years [18,31,32,33].

Given the critical role played by EPAC in multiple pathologies, several EPAC-specific modulators have been screened to target EPAC proteins (vide infra). Despite their wide commercial availability and seemingly ubiquitous use in experimental studies, for several EPAC inhibitors, the molecular basis of their interactions with EPAC and the related mechanism of action are currently not fully understood, thus limiting further improvement on existing inhibitor design, lead optimization and EPAC-targeted drug development.

Another notable impediment in drug discovery and development [34,35] arises from the hydrophobic nature of the aforementioned EPAC inhibitors, which makes them susceptible to the formation of colloidal aggregates in a hydrophilic environment. These aggregation prone-compounds can modulate specific enzyme-substrate interactions due to non-specific enzyme-aggregate absorption, ultimately modifying enzyme activity due to protein misfolding/unfolding, decrease in free substrate concentration, alteration of effective enzyme-substrate affinity and/or physical separation of the enzyme and substrate [34,35,36]. Overall, the phenomenon of non-specific enzyme inhibition is typically referred to as aggregation-prone inhibition (ABI) and is notorious for introducing false positives in drug screens [34,37] as well as posing several challenges in terms of dosage optimization and drug delivery in physiological systems.

Many of the aforementioned EPAC inhibitors have shown great therapeutic potential in cell-based and in vivo models despite their propensity to form colloidal aggregates [38,39,40,41]. Interestingly, several drug-like small molecules, lead compounds and even marketed drugs show aggregation-prone tendencies, particularly at micromolar concentrations [42], raising the question of how the specific interactions between enzymes and enzyme-specific inhibitors are affected by the latter’s aggregation-prone tendencies. Moreover, it has not been conclusively determined if all ligand-aggregates involved in ABI bind enzymes in the first place. Thus, a comprehensive understanding of the mechanism of ABI as well as the underlying specific enzyme:inhibitor interactions is critical to further optimize EPAC inhibitors.

As ABI is ubiquitous, several strategies have been implemented to counter aggregate formation, mainly exploiting the sensitivity of ligand aggregates to non-ionic detergents and other solubilizing agents [37,43]. As such, the detergent Triton X100 (TX) and human or bovine serum albumin (HSA or BSA) are commonly used to attenuate false positives associated with ABI in drug screens. However, they introduce the added risk of generating false negatives [34,44]. Therefore, in addition to understanding the basis of ABI when evaluating the therapeutic potential of EPAC-specific inhibitors (ESIs), if attenuators are used, awareness of the mechanism of ABI attenuation is also crucial to carefully define and consider the false positive vs. false negative balance.

The objective of this review is to summarize both the specific interactions underlying EPAC inhibition by two promising EPAC-specific inhibitors—CE3F4R [45] and ESI-09 [46]—as well as the non-specific interactions that result from their colloidal aggregate formation and subsequent aggregation-based EPAC inhibition in aqueous systems [47]. In this manner, we hope to provide key biophysical insight to the EPAC community in evaluating the inhibitory potential of current commercially available aggregation-prone EPAC inhibitors, in optimizing existing lead compounds, and in informing the future development of EPAC-specific inhibitors. Additionally, the specific mechanism of ABI attenuation by HSA and TX is also briefly discussed in the context of EPAC with the aim of advocating their conscientious use in screening ESIs and to recommend the careful consideration of false positive vs. false negative balance when evaluating inhibitor performance in the presence of such compounds.

## 2. cAMP-Dependent Regulation of EPAC Function

**Figure 1:**
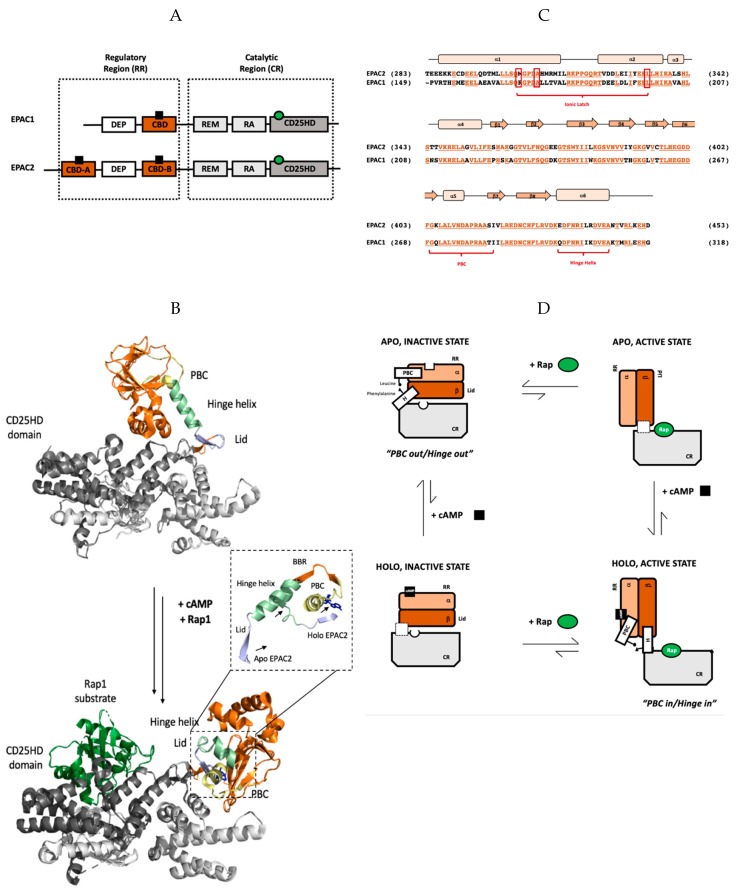
Structure and thermodynamics of the cAMP binding domain (CBD)-binding domain of EPAC. (**A**) Domain organization of Exchange Protein directly Activated by cAMP (EPAC) isoforms 1 and 2 with catalytic and regulatory regions indicated. cAMP is shown as a black square bound to the CBDs (highlighted in orange) in the regulatory region and the Rap protein substrate is represented by a green circle bound to the CD25 homology domain (colored dark grey), which is responsible for EPAC’s GEF activity. EPAC2 has an additional N-terminal CBD that is dispensable in the regulation of EPAC’s catalytic activity in response to cellular cAMP levels. (**B**) Global changes in EPAC2 structure upon cAMP binding. In both the apo, inactive state (top) and holo, active state (bottom), unless otherwise specified CBD-B is shown in orange and the catalytic region is shown in grey. Additionally, the PBC, hinge helix, lid region, and CD25HD domain are indicated in yellow, pale green, light blue, and steel grey, respectively; the Rap1 substrate protein is shown in dark green bound to the holo active state and the ligand Sp-cAMPS is indicated in dark blue. Only residues common to both crystal structures are shown in this panel. The zoomed-in view shows the aligned PBC, BBR, hinge helix, and lid of EPAC2 in the unbound apo, inactive state and the Sp-cAMPS bound holo, active state. The direction of movement of the PBC, hinge helix and lid upon ligand binding is represented by arrows. (**C**) Sequence alignment of the regulatory cAMP-binding domain from EPAC1 and EPAC2 with conserved residues colored in vermillion and underlined. The secondary structure elements (α-helices and β-sheets) are indicated by peach rectangles and orange arrows, respectively. Important structural motifs, namely the PBC, hinge helix and ionic latch, are labelled in red. (**D**) Schematic representing the four-state thermodynamic cycle of EPAC auto-inhibition and activation in response to cAMP-binding. cAMP shown as a black square and Rap GTPase as green circles; the phosphate-binding cassette and hinge helix are labelled PBC and H, respectively. Being transient species, the EPAC apo, active and holo, inactive states have not been isolated, thus the relative conformation of the PBC and hinge helix remain unknown. As such, no indication of the relative conformations of the PBC and hinge helix is reported in the diagram. Instead, this conformational uncertainty is represented by a dashed white box in place of the PBC and hinge helix in the respective states.

Prior to discussing the specific mechanism of EPAC inhibition by CE3F4R and ESI-09, it is essential to consider the architecture of EPAC and its contribution to the spatial and temporal regulation of cAMP-dependent signaling. EPAC is a single-chain, monomeric protein and both of its isoforms—EPAC1 and EPAC2—are composed of two regions: the N-terminal regulatory region (RR) and the C-terminal catalytic region (CR) (Figure 1A) [17,48,49]. The regulatory region consists of the disheveled-Egl10-plekstrin domain, which targets EPAC to specific subcellular membrane sites, and a cAMP binding domain (CBD), which allosterically regulates EPAC’s GEF activity [15,43,44]. Unlike EPAC1, EPAC2’s RR includes two CBDs of which CBD-B is homologous to that of EPAC1 (Figure 1A) and is required for cAMP-dependent activation [17,48,49], while CBD-A is non-essential for EPAC regulation, but it may serve a role in determining intracellular localization [50]. On the other hand, the organization of the CR in both isoforms is very similar, spanning the Ras-exchange motif (REM), the Ras association domain (RA), and a classic CD25 homology domain (CD25HD)—a structural element that catalyzes the GDP–GTP exchange on Rap GTPases that propagates the cAMP signal [17,48,49]. It is important to note that in both EPAC isoforms, cAMP-dependent activation depends on the relative conformation of the conserved N-terminal regulatory region (RR) to the C-terminal catalytic region (CR). When EPAC adopts its apo inactive state in the absence of cAMP, the CR and RR sample primarily a closed conformation, preventing Rap1 and Rap2 binding to the CR, effectively inhibiting EPAC GEF activity. When cellular cAMP levels rise, cAMP-binding to the conserved CBD of the RR acts as a molecular switch, allowing EPAC to assume an open topology, which enables Rap access to the CR and facilitates subsequent guanine nucleotide exchange (Figure 1B).

The EPAC1 CBD and the EPAC2 CBD-B are homologous, evolutionarily conserved structural elements that trigger the aforementioned conformational change, thereby acting as key modulators of cAMP-dependent signaling. As seen in Figure 1C, the CBD of both isoforms is composed of an α-helical subdomain consisting of six α-helices punctuated by a β subdomain spanning eight β sheets [16,48,49]. The α-1 and α-2 helices of the N-terminal α-subdomain form multiple salt-bridges with the catalytic region in the closed topology. These interactions constitute the ionic latch (IL), which blocks Rap access to the CR, maintaining constitutive inhibition [51,52,53,54]. Helices α2—4 form a conserved structural element denoted as N3A, which is found in all cis-regulated CBDs [55]. The β subdomain, on the other hand, contains two main regions for cAMP-binding, namely the base-binding region (BBR) spanning β4 to β5, as well as the phosphate-binding cassette (PBC), an indispensable region also for eliciting the allosteric effects of cAMP-binding [17,48,49]. At the C-terminus of the RR, the hinge helix or α6 serves as another crucial structural element of EPAC auto-inhibition in the absence of cAMP and of cAMP-dependent EPAC activation (Figure 1B,C) [17,48,49,51,52].

The concerted movements of the PBC and hinge helix shift the protein conformation between the open and closed topologies, and therefore underlie cAMP-dependent EPAC activation. In the absence of cAMP, the PBC shifts “outward”. To maintain a crucial hydrophobic contact between conserved Leucine in the PBC and a Phenylalanine in the hinge helix, the latter also adopts the “out” conformation [51,56,57,58]. As a result, the region C-terminal to the hinge helix, also known as the “lid”, moves forcing the RR to occlude the Rap-binding site of the CR’s CD25HD, i.e., EPAC adopts the closed topology characteristic of its apo, inactive state [51,56,57,58]. Upon cAMP-binding, the PBC shifts “inward” and the CBD equilibrium shifts from the inactive state to the active state, wherein the hinge helix also moves inward to maintain the hydrophobic contact with the PBC, moving the lid and allowing Rap protein substrates to access the CR in the open topology (Figure 1B,D) [51,56,57,58]. Upon removal of cAMP, the PBC and hinge regain the out orientation [51,56,57,58]. In thermodynamic terms, the cAMP-binding and EPAC inhibition equilibria couple to form a four-state cycle (Figure 1D), which provides a general framework to analyze the mechanism-of-action of EPAC-targeted inhibitors, as discussed below in the context of CE3F4R and ESI-09.

## 3. The Discovery of EPAC-Specific Inhibitors

The existence of two ubiquitously expressed cAMP-dependent sensors, EPAC and PKA, necessitates the development of selective pharmacological interventions to modulate their discrete functions. Such EPAC vs. PKA selectivity was obtained for the first time through structure-based design, which led to the identification of a cAMP analog that functions as an activator for EPAC but not PKA [59]. However, in contrast to EPAC agonists, identifying EPAC selective antagonists has proven much more challenging, as cyclic nucleotide analogues often interfere with phosphodiesterase activities, which increases cellular cyclic nucleotide levels and counters the intended antagonistic effects.

In 2012, a major breakthrough in developing EPAC-specific small molecule inhibitors was reported with the development of a robust EPAC-based high-throughput screen (HTS) assay [60]. This effective EPAC HTS assay utilized a fluorescence cAMP analogue—8-NBD-cAMP—to rapidly identify hits that compete with cAMP binding to EPAC2. Implementation of this simple “mix-and-read” assay led to the discovery of a panel of EPAC specific inhibitors (ESIs) starting from a 14,400 drug-like compound library [60,61]. Two such compounds, ESI-05 and ESI-07, are exclusive for EPAC2 as they bind the interface between the EPAC2 CBD-A and CBD-B, locking the enzyme in its auto-inhibitory conformation [60,61]. However, the majority of the identified ESIs exhibit pan-EPAC activity, of which one particular compound ESI-09 has been a significant research focus in recent years.

The use of a functional fluorescence-based Rap1 nucleotide exchange assay led to the identification of another EPAC-specific inhibitor from a screen of 640 compounds, namely CE3F4 [62]. Unlike ESI-09, a competitive inhibitor, CE3F4 inhibits EPAC in an uncompetitive fashion [62]. Being an N-formyl compound, CE3F4 exists as interconverting rotamers, which populate an inseparable 3:1 *E*:*Z* mixture [62,63]. Although the major rotamer predominates in DMSO solution as shown by NMR studies, crystallography studies revealed that the minor rotamer exhibits better packing [63]. The CE3F4 stereochemistry is also critical. Importantly, (*R*)-CE3F4 is more potent than the racemic CE3F4 and (*S*)-CE3F4, and exhibits approximately 10-fold higher selectivity for EPAC1 over EPAC2 [64]. Structure activity relationship studies later identified the two bromine atoms on the phenyl ring and the formyl group as critical for EPAC1 selective inhibition [63].

Subsequently, Brown et al. identified non-competitive EPAC1 inhibitors from a virtual screen using a diverse compound library (Chembridge) [65,66]. A follow up 8-NBD-cAMP-based HTS assay using isolated CBD domains of EPAC1 or EPAC2 led to the identification of partial agonists [67]. An arylsulfonamide I942 was found to act as a partial agonist for EPAC1 with an apparent AC_50_ value of ~40 μM and a maximal activity of ~10% compared to cAMP [67].

The identification of EPAC specific inhibitors via HTS campaigns and subsequent medicinal chemistry optimizations have provided a set of useful ligands for interrogating EPAC mediated cell signaling. In particular, ESI-09 exhibits excellent in vivo pharmacological and toxicological profiles and has demonstrated therapeutic efficacy in various preclinical animal models [38,39,68]. These developments establish EPAC proteins as promising therapeutic targets. Hence, it is paramount to understand the mechanisms underlying both specific and non-specific interactions of EPAC modulators, as discussed here in the context of CE3F4R and ESI-09.

## 4. Specific and Non-Specific Inhibition of EPAC1 by CE3F4R

Classical uncompetitive inhibitors specifically target the enzyme-substrate complex as opposed to the free-enzyme, thus there is no binding competition with the substrate [69,70]. Increasing substrate concentration amplifies the effectiveness of the inhibitor. Furthermore, sole recognition of the enzyme:substrate complex instead of the free enzyme increases the selectivity of binding relative to competitive inhibitors. As such, uncompetitive inhibition allows for simultaneous optimization of both binding specificity and inhibitory potency and is therefore an appealing strategy for pharmacological and biological intervention [69,70].

**Figure 2:**
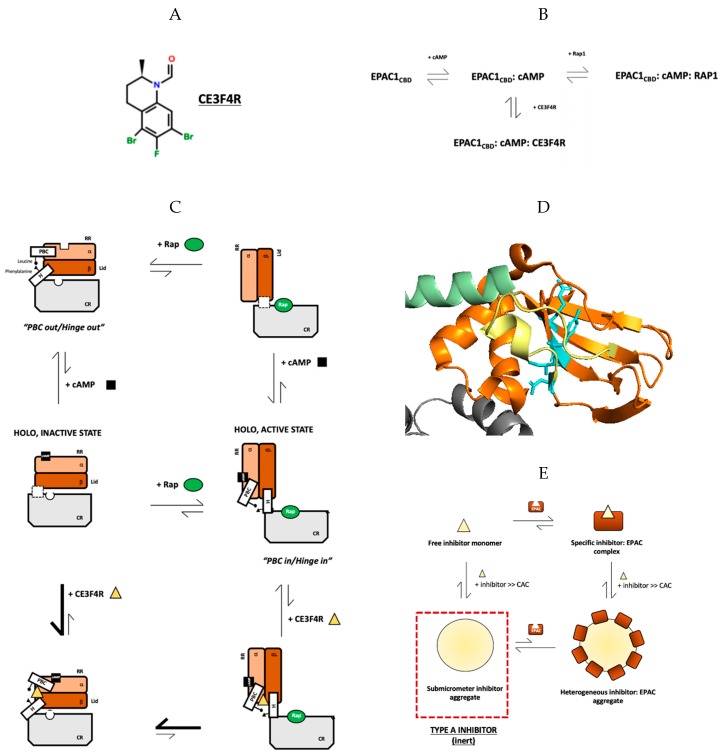
Specific and non-specific interactions of EPAC1_CBD_ and CE3F4R, a novel uncompetitive inhibitor. (**A**) The molecular structure of CE3F4R. (**B**) Schematic representing the uncompetitive mechanism of EPAC1 inhibition by CE3F4R. (**C**) Schematic summarizing the perturbation of the classic four-state thermodynamic cycle of EPAC activation by cAMP by CE3F4R binding, particularly highlighting the stabilization of the mixed holo inactive intermediate with the phosphate-binding cassette (PBC) in the active and hinge helix in the inactive conformation. Relative conformations of the PBC and hinge helix have not yet been elucidated in the holo, inactive and apo, active states and are thus not shown (**D**) Specific binding site of CE3F4R at the α/β subdomain interface of EPAC1 including residues Y242, I243, D267, and R294, as indicated in cyan, at the β-sheet facing the α-subdomain; the image shows homologous residues in EPAC2. Color scheme followed is consistent with Figure 1B. (**E**) Proposed thermodynamic cycle encompassing both specific enzyme:inhibitor binding as well as non-specific interactions between the two species as a result of colloidal aggregate formation; CE3F4R, as indicated on the figure, is a type-A inhibitor, forming inert aggregates that do not interact directly with the protein. Instead, they reduce overall inhibitory effect by acting as sinks for monomeric inhibitors (Figure adapted from Boulton, S.; Selvaratnam, R.; Ahmed, R.; Van, K.; Cheng, X.; Melacini, G. Mechanisms of specific versus nonspecific interactions of aggregation-prone inhibitors and attenuators. *J. Med. Chem.* **2019**, *62*, 5063–5079. Copyright (2019) American Chemical Society).

Upon its discovery, CE3F4R (Figure 2A) was confirmed to act as an unconventional uncompetitive inhibitor, being unable to appreciably inhibit EPAC1’s catalytic activity upon the removal of the CBD. This observation suggests that CE3F4R did not bind the substrate-specific site or any other site in the CR, as instead expected for classical uncompetitive inhibition [62,64]. Whereas classical uncompetitive inhibitors are selective for the E:S complex, non-classical uncompetitive inhibitors are selective for the enzyme:allosteric effector complex [62,64]. CE3F4R belongs to the latter class as it specifically inhibits cAMP-bound EPAC rather than apo EPAC, thus forming an EPAC1:cAMP:CE3F4R ternary complex (Figure 2B).

Boulton et al. elucidated the mechanism of specific inhibition of EPAC1 by CE3F4R, employing a diverse array of biomolecular NMR approaches supplemented by various other techniques [45,71,72,73]. To gain insight into the nature of the EPAC1 inhibitory conformation within the EPAC1:cAMP:CE3F4R ternary complex, NMR chemical shift projection analyses (CHESPA) were performed to measure the degree of residue-specific fractional activation (X). The X values report on the extent to which each residue in the EPAC1_CBD_:cAMP:CE3F4R ternary complex resembles the inactive apo CBD (X = −1) vs. the active CBD:cAMP complex (X = 0) [74,75,76,77]. Using CHESPA, the hinge helix was identified as significantly shifted towards inactivation (X ≈ −0.5), together with the adjacent α4 helix, but the PBC did not show appreciable changes compared to the cAMP-bound holo, active state (X ≈ 0) [45]. Thus, CE3F4R binding does not appear to shift the conformation of EPAC from the “PBC in/hinge in” holo, active state to the “PBC out/hinge out” apo, inactive state. Rather, it seems to stabilize a mixed intermediate, in which the hinge conformation is apo-like, but the PBC is holo-like, in a similar “in” conformation as the cAMP-bound state (Figure 2C) [45].

Further support for the notion that CE3F4R targets the PBC in-hinge out mixed intermediate, came from studying the interaction of CE3F4R with the EPAC L273W variant, wherein the Tryptophan’s bulky indole group sterically hinders the hydrophobic contact between the PBC and the hinge helix [45]. As a result, this variant is locked in the “PBC in, hinge out” mixed conformation. Consistent with the prediction of CE3F4R’s preferential binding to this mixed state, a five-fold increase in CE3F4R binding affinity was observed for the L273W mutant compared to the wildtype [45].

Finally, negligible binding was observed when CE3F4R was added to Rp-CAMPS bound EPAC1. The binding of Rp-cAMPS reverts the enzyme completely to its apo, inactive state, effectively destabilizing the mixed intermediate [45,78]. Negligible CE3F4R binding to the resultant “PBC out/hinge out” conformation served as a negative control, further validating the conclusion that the CE3F4R’s mechanism of uncompetitive inhibition involves stabilizing the cAMP:CE3F4R:EPAC1_CBD_ ternary complex in a mixed intermediate in which the PBC remains “in”, while the hinge helix adopts the “out” conformation (Figure 2C). This inhibitory mechanism is somewhat reminiscent of the stabilization of a mixed intermediate of PKG by cAMP, which acts as a partial agonist for this kinase [79,80].

In addition to determining the allosteric effects of CE3F4R binding, Boulton et al. also went further and defined the specific CE3F4R binding site [45]. Residues involved in CE3F4R binding were initially screened using CHESPA based on the non-linearity of their projection vectors relative to the reference (cosθ < 0.9) [45]. As predicted by the simple CCS magnitude map, all the residues were found to be clustered at the α/β subdomain interface [45]. These findings were independently validated by saturation transfer difference (STD) experiments with a ^13^C^1^H-HSQC spectral read out and with saturation of the inhibitor’s formyl resonance [75,76,77,81]. The residues with significantly higher STD/STR ratios were overall consistent with those that exhibited the highest degree of non-linearity in the CHESPA analysis. Residues involved in CE3F4R binding were thus identified as Y242, I243, D267, and R294 (Figure 2D) [40]. Using a paramagnetic relaxation enhancement (PRE) [82,83] experiments called SLAPSTIC or spin-labels attached to protein side chain as a tool to identify interacting compounds, together with a triangulation methods based on two different spin-label locations, the proposed binding site at α/β subdomain interface was independently confirmed [45]. Thus, the specific CE3F4R binding site was concluded to span at least four key residues on the α/β subdomain interface of EPAC1’s RR [45].

Boulton et al. also elucidated the basis of CE3F4R selectivity for EPAC1 vs. EPAC2 [45]. By probing the α/β subdomain interface, two main differences were found between the two isoforms: W245 (L380 in EPAC2) and Q270 (K405 in EPAC2) [45]. The latter residue is especially important in maintaining and stabilizing the holo, active state in EPAC2 by forming a salt bridge with E443—an interaction that stabilizes the hinge helix in the “in” conformation, thereby reducing the population of the “PBC in, hinge out” intermediate and, in turn, the likelihood of CE3F4R binding [45]. This Lysine-Glutamate salt bridge was found to be the basis of CE3F4R selectivity by creating the EPAC1 Q270K mutant, observing chemical shift changes in the NH-HSQC spectra and performing CHESPA [45]. Both the mutant apo- and cAMP-bound states exhibited a shift toward activation for the hinge helix and showed significantly reduced chemical shift changes upon CE3F4R addition, confirming that the K/E salt bridge in EPAC2 and resultant stabilization of the “hinge in” conformation is the major determinant for the selectivity of CE3F4R for EPAC1 vs. EPAC2 [45].

Although CE3F4R is a promising candidate for therapeutic applications due to its uncompetitive inhibition mechanism and selectivity for EPAC1, it is prone to aggregation due to its hydrophobicity. This property may limit the maximum CE3F4R concentration used in therapeutic applications and increases susceptibility to non-specific effects such as aggregation-based inhibition. This led Boulton et al. to investigate CE3F4R’s non-specific interactions with EPAC1 using diverse techniques with varying degrees of resolution [47]. The formation of CE3F4R aggregates in vitro was studied using two commonly-used techniques, namely dynamic light scattering (DLS) and transmission electron microscopy (TEM) [34,35,42,47]. With both techniques, sub-micrometer aggregates were observed with sizes between 60 to 900 nm [47]. According to TEM, however, unlike most aggregation-prone inhibitors, CE3F4R aggregates display a more varied, irregular morphology [34,35]. The critical aggregation concentration (CAC), a key determinant of the onset of non-specific interactions, was determined to be ~150 μM by monitoring the point of deviation from linearity in the proportional increase in ^1^H NMR peak intensities with increasing inhibitor concentrations [47]. This value was independently validated using STD experiments, where STD signals appeared at CE3F4R concentrations above 150 μM—testament to the formation of high molecular weight aggregates [47].

After establishing that CE3F4R, like most prototypical hydrophobic drug leads, formed large, colloidal aggregates at micromolar concentrations, Boulton et al. also determined the mechanism of CE3F4R’s ABI and its non-specific interactions with EPAC1. As opposed to the commonly observed ABI phenomenon where the protein receptor is non-specifically adsorbed onto the aggregate surface [34,35,42], CE3F4R aggregates did not interact directly with EPAC1. In the CE3F4R titration, when the inhibitor concentration was increased significantly beyond the CAC, HN-HSQC cross-peaks shifted from the inactive, CE3F4R-bound state to the active CE3F4R-unbound state, likely representing a dissociation event in which the CE3F4R aggregates recruit CE3F4R out of the EPAC1:cAMP:CE3F4R ternary complex [47]. As EPAC1 binds CE3F4R, it reduces the free inhibitor concentration, increasing the total inhibitor concentration needed for aggregate formation and for the dissociation event to occur [47]. Moreover, since no intensity losses were observed in the EPAC1 HSQC spectra despite aggregate formation, it was confirmed that EPAC1 did not adsorb or form a high molecular weight complex with the aggregates. This conclusion was supported by DLS experiments in which the EPAC1 volume profile with a dominant 4.2 nm peak did not deviate significantly upon the addition of inhibitor at concentrations above the CAC [47]. Thus, CE3F4R was classified as a type-A aggregation-prone inhibitor, which forms inert aggregates that do not adsorb EPAC1, but rather interfere with the specific enzyme:ligand binding by acting as a sink for the monomeric inhibitor (Figure 2E) [47].

By reducing free inhibitor concentration, the non-specific interactions encompassing type-A ABI compete with specific enzyme:inhibitor interactions. Thus, instead of a standard sigmoidal monotonic dose response curve, a bell-shaped bi-modal dose response curve in which the maximum therapeutic efficacy occurs at intermediate concentrations is expected for type-A inhibitors [84,85]. As aggregation-based inhibition involves the coupling of specific binding and aggregation equilibria, the key determinants of this interaction are the K_d_ of the specific enzyme:inhibitor interaction and the CAC [47]. As [I] > 10* K_d_ is required for ~90% saturation of the enzyme’s specific binding sites, if the CAC is less than 10* K_d_, then the enzyme cannot be fully saturated without non-specific ABI effects becoming significant. Thus, to gauge their applicability as a pharmacological intervention for EPAC-related pathologies, it is critical to determine the optimal therapeutic window of concentrations for type-A inhibitors.

## 5. Specific and Non-Specific Inhibition of EPAC1 by ESI-09

**Figure 3:**
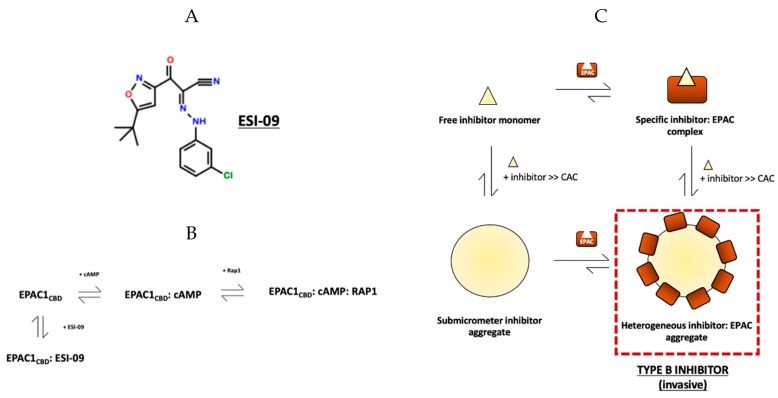
Specific and non-specific interactions of EPAC1_CBD_ and ESI-09, a competitive inhibitor. (**A**) The molecular structure of ESI-09. (**B**) Schematic representing the competitive mechanism of EPAC1 inhibition by ESI-09. (**C**) Proposed thermodynamic cycle summarizing specific enzyme:ESI binding in addition to non-specific interactions between the two species as a result of aggregation; ESI-09, as indicated on the figure, is a type-B inhibitor, forming invasive aggregates that non-specifically adsorb protein molecules and may subsequently decrease specific enzyme:ESI-binding by causing protein misfolding, sequestering protein from ligand, as well as other diverse mechanisms (Figure adapted from Boulton, S.; Selvaratnam, R.; Ahmed, R.; Van, K.; Cheng, X.; Melacini, G. Mechanisms of Specific versus Nonspecific Interactions of Aggregation-Prone Inhibitors and Attenuators. *J. Med. Chem.* **2019**, *62*, 5063–5079. Copyright (2019) American Chemical Society.).

Along with CE3F4R, ESI-09 (Figure 3A) has also garnered significant attention in recent years as a promising EPAC-targeted therapeutic with aggregation-prone tendencies [86]. ESI-09 exhibits pan-EPAC inhibitory effects in in vitro and in vivo studies, where it was also found to hinder the progression of pancreatic cancer [24], prevent bacterial invasion in fatal rickettsioses [68] and regulate T-cell mediated suppression of the immune response [87]. Despite these promising results, at high concentration (>25 μM), ESI-09 perturbs cAMP-independent exchange functions of EPAC2 as well as decreases protein stability, suggesting that ESI-09 under these conditions is a non-specific protein denaturant [88]. However, recently, a comprehensive investigation by Zhu et al. into the structure/activity relationship (SAR) and the biophysical basis of ESI-09 action demonstrated that ESI-09 is indeed a competitive inhibitor that interacts specifically and selectively with EPAC (Figure 3B) [46].

The specific interactions involved in ESI-09’s inhibitory mechanism were elucidated using diverse experimental methodologies. First, the 3-chlorophenyl moiety on ESI-09 (Figure 3A) was found to confer specificity to ESI-09 based on SAR analyses [46]. By screening a series of compound with the core ESI-09 structure (Figure 3A) and varying mono- and di-substituted chlorobenzene rings, it was found that 3- and 5-chlorosubtituted rings interact most favorably with EPAC2 and therefore had the highest affinity for EPAC2 [46]. The competitive nature of EPAC inhibition by ESI-09 was then independently confirmed using a competition assay with fluorescent BODIPY-GTP, an EPAC substrate, where a dose-dependent decrease in cAMP-mediated GTP/GDP exchange was observed [46]. Additionally, increasing cAMP concentration was found to completely reverse ESI-09’s inhibitory effect, consistent with competitive inhibition. Thus, ESI-09 was concluded to interact specifically with EPAC1 and EPAC2 as a competitive specific inhibitor with an apparent IC_50_ of 4.4 μM for EPAC2 in the presence of 20 μM cAMP [46].

The previous claim that ESI-09 causes general protein denaturation was then addressed explaining that at [ESI-09] > 25 μM the general hydrophobic nature of ESI-09 results in the formation of colloidal aggregates that non-specifically adsorb proteins [46]. The specificity of ESI-09:EPAC interactions was independently confirmed using NH-HSQC spectra, which exhibit well-dispersed peaks even in the presence of ESI-09—a defining characteristic for fully-folded proteins—with intensity losses for selected residues, likely due to intermediate exchange [46]. However, increasing ESI-09 inhibitor concentrations beyond 500 μM resulted in line-broadening of almost every HSQC peak beyond detection, which was consistent with non-specific adsorption of the protein into ligand aggregates and the formation of high molecular weight NMR-invisible species [46]. In short, Zhu et al. provided considerable evidence that ESI-09 interacts specifically with EPAC as a competitive inhibitor, and highlighted a potential limitation for the use of ESI-09 in enzymatic assays at high concentrations—aggregate formation and subsequent aggregation-based inhibition.

Building on Zhu et al.’s work, the mechanism of non-specific EPAC inhibition by ESI-09 was also determined by employing biomolecular NMR techniques in combination with other biophysical techniques with varying degrees of resolution [47]. First, aggregate formation by ESI-09 was documented and confirmed using DLS and TEM, which showed that much like CE3F4R, ESI-09 also formed submicrometer aggregates in aqueous buffers with a size range from 90–600 nm [47]. However, ESI-09 aggregates displayed a more regular, circular morphology similar to that of traditional aggregation-prone compounds [34,35,42,47]. Next, the CAC of ESI-09 was determined by monitoring the ^1^H NMR signal line widths and conducting STD experiments, as described earlier, to be ~150 μM [47]. Thus, Boulton et al. confirmed using DLS, TEM and NMR data that ESI-09 forms large colloidal aggregates in aqueous solutions.

Importantly, the mechanism of ABI of EPAC by ESI-09 is in stark contrast to CE3F4R. Whereas CE3F4R forms inert, type-A aggregates, ESI-09 acts as a more traditional aggregation-prone inhibitor, forming aggregates that non-specifically adsorb proteins (Figure 3C) [47]. Evidence for this conclusion came from observing the NH-HSQC spectra from an EPAC titration with ESI-09. At concentrations below the CAC, the spectra showed intensity losses for selected residues, likely due to intermediate exchange broadening—a characteristic of specific binding [47]. Chemical shift perturbations were then used to calculate the K_d_ of ESI-09 for the EPAC1_CBD_ to be 20 μM, which was then confirmed with a competitive binding experiment with 8-NBD-cAMP—a fluorescently labelled cAMP molecule [47]. Unlike CE3F4R, when the ESI-09 concentration was increased well above the CAC, the signal losses became significantly more pervasive and pronounced, indicating that EPAC1_CBD_ interacts with ESI-09 aggregates [47]. This finding was corroborated by comparing through DLS the size profile of EPAC1_CBD_ with the addition of ESI-09 below and above its CAC, which revealed a significant size increase in the latter case from 4.2 nm diameter of the apo protein to ~9 nm [47]. As 9 nm is also significantly smaller than the 250 nm mean diameter of ESI-09 aggregates, this measurement was likely the population-weighted average of free and aggregate-bound EPAC1_CBD_ [47]. Boulton et al. ultimately classified ESI-09 as a type-B inhibitor which forms promiscuous colloidal aggregates at concentrations above the respective CAC (Figure 3E) [47].

Although different in terms of ABI mechanism from type-A inhibitors, type-B inhibitors also affect enzyme:substrate binding and dynamics but by non-specifically recruiting proteins into their sub-micrometer aggregates and, in the case of ESI-09, by possibly unfolding the protein [47]. In this manner, both type-A and type-B inhibitors interfere with drug screening and characterization. Hence, their respective K_d_ and CAC values must be determined in order to draw conclusions about their potency and specificity at various concentrations—crucial parameters in later determining the pharmacological value of the drug lead. Unlike CE3F4R, the K_d_ for the EPAC1_CBD_ and CAC values of ESI-09 are known to be 20 μM and ~150 μM, respectively [47]. Based on this information and the assumption of a similar K_d_ for the full-length EPAC, for >90% enzyme saturation and maximum inhibitory potency, an ESI-09 concentration greater than 200 μM is needed, which is greater than the CAC value. However, in in vivo studies, ESI-09 effectively ameliorates pathological conditions and exhibits excellent bioavailability with minimal toxicity at concentrations less than 20 μM, i.e., less than the K_d_ [46]. Based on these observations, Zhu et al. defined the therapeutic window for ESI-09 to be at a concentration between 1–10 μM which is below the CAC, and considered pharmacologically effective [24,26,68,87,89]. Although the presence of ESI-09 aggregates within cells is currently unclear, the aggregation of ESI-09 and other hydrophobic drug leads causes assay interferences in vitro and limits the maximum inhibitor concentration at which specific interactions prevail. Hence, diverse strategies for ABI attenuation should be developed to increase the specificity and potency of inhibitor effects in aqueous buffers.

## 6. Attenuation of Aggregation-Based Inhibition Caused by EPAC-Specific Inhibitors

Traditional strategies for attenuating aggregation-based inhibition capitalise on the aggregate’s sensitivity to non-ionic detergents, particularly Triton X100 (TX), and other solubilizing agents, such as human or bovine serum albumin (HSA) [43,90,91]. Historically, both approaches have been extensively utilized to eliminate non-specific binding in drug screening and biochemical assays. Their ability to disrupt ABI for well-known promiscuous inhibitors led to wide-spread utilization as tools to diagnose and eliminate ABI for almost the past two decades. However, a recent study has raised concerns about the potential of these ABI attenuators to disrupt target-specific, ABI-independent interactions [47]. For example, the hydrophobic core of TX micelles may recruit and sequester free, hydrophobic inhibitors. Even when TX concentrations do not exceed its critical micellar concentration (CMC) [37,92], there is the possibility that it will co-aggregate with hydrophobic compounds and limit their ability to interact with their intended targets. HSA, on the other hand, is a transport protein that interacts with a plethora of hydrophobic ligands, and, as such, has the ability to potentially interact and sequester hydrophobic compounds being screened as inhibitors [93,94]. In short, TX and HSA can compete with specific enzyme:inhibitor binding, diminishing the extent of specific enzyme inhibition and introducing potential false-negatives [47]. Thus, it is important to understand the molecular mechanism of ABI attenuation by TX and HSA and consider the false positive vs. false negative balance when employing such compounds in drug screens as well as in vitro and possibly in vivo studies.

Understanding ABI attenuation is especially pertinent when evaluating EPAC-targeted strategies as the majority of the currently known EPAC antagonists are small hydrophobic compounds that are aggregation-prone in aqueous buffers. Along with elucidating the non-specific interactions underlying ABI of EPAC by CE3F4R and ESI-09, Boulton et al. also determined the mechanism of ABI attenuation by TX and HSA as well as the nature of their interactions with EPAC1_CBD_ and the EPAC-specific inhibitors [47]. First, TX interactions with EPAC1_CBD_ were monitored by NH-HSQC spectral analysis at 0.1% concentration—well above the CMC [95]—and no significant chemical shift changes or line broadening was observed, indicating that TX micelles are inert with respect to EPAC1_CBD_-binding and do not facilitate enzyme unfolding [47]. Next, interactions between TX and the ESIs were studied at various concentrations of both species. At concentrations, significantly below the CMC for TX and CAC for CE3F4R and ESI-09, the formyl moiety of CE3F4R was found to interact with TX, facilitating the formation large co-aggregates [47]. Similarly, ESI-09 also formed heterogeneous co-aggregates, as detected by ^1^H line broadening. However, based on DLS size profiles, the size and morphology of the CE3F4R:TX and ESI-09:TX co-aggregates were distinct—when 1:1 concentrations are used, the CE3F4R:TX co-aggregate population is unstable and heterogeneous with respect to size, whereas ESI-09:TX co-aggregates have a more homogenous population [47]. Thus, it was inferred that TX micelles were capable of incorporating various hydrophobic inhibitors, effectively decreasing the free inhibitor concentration and reducing both non-specific and specific enzyme:inhibitor interactions.

**Figure 4:**
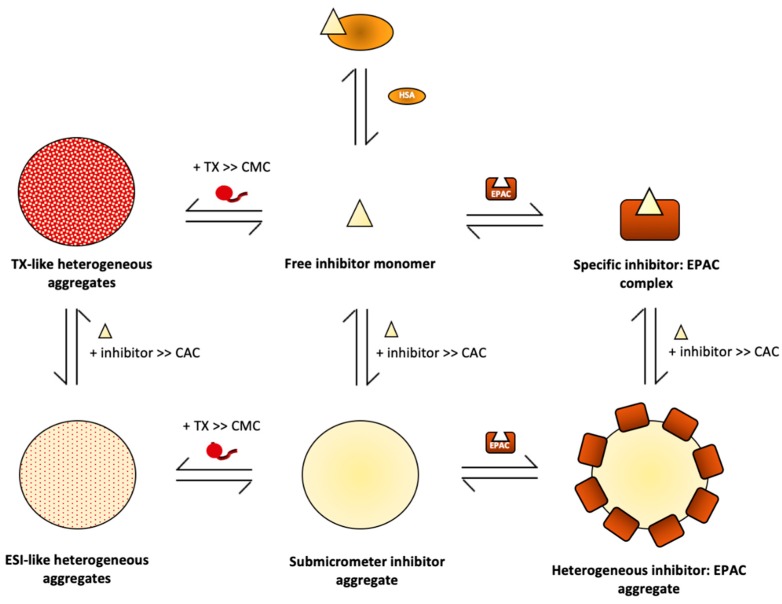
Specific and non-specific interactions of aggregation-prone inhibitors and aggregation attenuators in the context of EPAC. Aggregation-prone inhibition tends to decrease specific EPAC:ESI interactions. Inert type-A inhibitor aggregates do not interact with the protein receptor, however, they act as sinks for inhibitors, decreasing the free inhibitor concentration which ultimately reduces a compound’s specific inhibitory effect at a particular concentration. In contrast, invasive type-B inhibitor aggregates non-specifically adsorb protein molecules, directly interfering with enzyme:inhibitor interactions. To attenuate the effects of ABI, TX, and HSA are commonly used. HSA directly binds monomeric inhibitors, decreasing free inhibitor concentration, which protects the system from aggregate formation. On the other hand, TX binds ESI aggregates, forming heterogeneous co-aggregates that exhibit inert properties when [TX] >> [ESI] or possibly invasive properties when [ESI] >> TX. Overall, the key thermodynamic parameters needed to evaluate the relative role of specific binding, aggregation and attenuation are the dissociation constants for each of the aforementioned specific interactions, as well as the CAC of the inhibitor and the CMC of the attenuator. Figure adapted from Boulton, S.; Selvaratnam, R.; Ahmed, R.; Van, K.; Cheng, X.; Melacini, G. Mechanisms of Specific versus Nonspecific Interactions of Aggregation-Prone Inhibitors and Attenuators. *J. Med. Chem.* **2019**, *62*, 5063–5079. Copyright (2019) American Chemical Society.).

The aforementioned hypothesis was validated by Boulton et al. for both CE3F4R and ESI-09 [47]. The displacement of the type-A CE3F4R from its binding site in the EPAC_CDB_ was monitored to determine the effect of TX on specific inhibitor binding. Based on NH-HSQC analysis, the spectrum of EPAC1_CBD_ previously-bound to CE3F4R shifted to its CE3F4R-unbound state upon addition of TX, confirming the hypothesis that TX competitively extracts inhibitors from specific ESI complexes [47]. The addition of TX to EPAC1_CBD_ previously bound to ESI-09, a type-B inhibitor, at concentrations below its CAC also demonstrates the same effect [47]. Upon confirming that TX competitively interferes with specific EPAC:ESI binding, the protective effect of TX was measured in preventing the recruitment of EPAC1_CBD_ to ESI-09 aggregates at TX concentrations greater than its CMC and ESI-09 concentrations above its CAC [47]. Here, NH-HSQC intensity losses were still observed despite the addition of TX, suggesting that TX addition has no perceivable protective effect on EPAC1_CBD_ recruitment and non-specific adsorption to ESI-09 aggregates under these conditions [47]. However, using NMR approaches, the incorporation of TX into ESI-09 aggregates was observed, suggesting that the relative abundance of the two species in these co-aggregates may dictate its properties [47]. Specifically, if ESI-09 is the dominant species, the TX:ESI-09 micelles may display ESI-09’s characteristic promiscuity and further contribute to non-specific ABI of EPAC. Overall, although TX is a widely-used non-ionic detergent in aggregation-based drug screens, it can introduce several artifacts into assay results by acting as a competitive sink for free inhibitors, thereby introducing false negatives as well as being incorporated into ligand aggregates with invasive properties (Figure 4). Thus, the use of TX in such applications requires careful consideration to minimize the loss of specific vs. non-specific interactions.

The mechanism of ABI attenuation by HSA has also been examined. Using surface plasmon resonance (SPR), Boulton et al. confirmed that HSA disrupts ESI aggregation [47]. However, their NMR analysis also demonstrates that HSA leads to competition with EPAC for binding the free inhibitor, similar to TX [47]. Using a collection of NMR and fluorescent probes that selectively bind HSA at specific sites, Boulton et al. determined through competition experiments the binding sites, stoichiometries and affinities of the ESIs for HSA. The analysis revealed the ESIs bind HSA with affinities that are significantly greater than those for EPAC. The latter finding is particularly disconcerting, indicating that ESI-09 has higher affinity for HSA than for EPAC1_CBD_ which would significantly shift free inhibitor in the specific EPAC:ESI binding equilibrium towards HSA binding. As such, the use of HSA may have major implications in assays, increasing the likelihood of false negatives. SPR, DLS and HN-HSQC NMR peak intensities were then used to determine if the addition of HSA to inhibitors at concentrations above the CAC would significantly attenuate the magnitude of aggregate formation and this was observed to be the case with both CE3F4R and ESI-09 [47]. Although HSA increases the inhibitor concentrations required to reach similar fractional saturations of EPAC1_CBD_ in the HSA-free conditions, HSA significantly impedes both CE3F4R and ESI-09 aggregate formation [47]. Thus, it is a promising alternative strategy to TX in attenuating aggregation in drug screens, but the false positive vs. false negative balance should still be carefully considered when interpreting results from experiments employing HSA.

## 7. Conclusions

In this review, we have explored the biophysical basis of the specific and non-specific interactions of two prototypical hydrophobic EPAC inhibitors—CE3F4R and ESI-09. Using solution NMR methods, CE3F4R was found to serve as a non-classical uncompetitive inhibitor that selectively binds a mixed intermediate in the cAMP-dependent thermodynamic cycle of EPAC activation [45]. This mixed intermediate displays features of both the cAMP-bound holo, active state where the PBC is in the “in” conformation, as well as that of the cAMP-unbound apo, inactive state where the hinge helix is in the “out” conformation [45]. As a result, CE3F4R binding captures EPAC1 in its holo, inactive conformation and stabilizes its closed topology, effectively inhibiting the enzyme. The specific binding site for CE3F4R is the α/β subdomain interface in the EPAC cAMP-binding domain, which becomes available upon cAMP binding—further testament to the inhibitory mechanism being uncompetitive with respect to cAMP, an allosteric effector, rather than the substrate [45]. Additionally, CE3F4R binding is specific to EPAC1 instead of EPAC2 due to the key residue Q270 that is unique to the former isoform [45]. As CE3F4R uncompetitively and specifically inhibits EPAC1 upon cAMP-binding, a signaling mechanism under tight spatiotemporal regulation, it is a potent and highly selective EPAC-targeted therapy that may be used to slow down the progression of multiple pathologies by tracking the spatial and temporal cAMP gradients. Further studies should aim to define an in vivo therapeutic window for CE3F4R action, wherein specific binding is optimized and non-specific effects such as ABI are negligible. Additionally, CE3F4R can also serve as a model in designing a new class of EPAC non-classical uncompetitive inhibitors that target mixed intermediates, thereby maximizing both inhibitory specificity and potency.

On the other hand, ESI-09, the second inhibitor discussed, is a pan-EPAC competitive inhibitor that has demonstrated significant pharmacological effect in preventing the invasion and metastasis of pancreatic and breast cancers, as well as providing protection from fatal rickettsioses [24,68]. At concentrations below 20 μM, the K_d_ of the EPAC1_CBD_-ESI-09 interaction, ESI-09 acts as a competitive inhibitor. Thus, the therapeutic window for ESI-09 action is defined at concentrations below 20 μM, ideally between 1–10 μM, which are considered pharmacologically effective, however, are not conducive to inhibitor aggregation and subsequent ABI [46,47].

Due to their hydrophobic nature, both CE3F4R and ESI-09 display aggregation in aqueous systems, which is a significant impediment in both the reliable characterization of new drug leads as well as in their effective administration and use. Despite their similar physical properties, CAC and aggregate sizes, CE3F4R and ESI-09 form aggregates with distinct morphologies and protein-binding behavior [47]. First, CE3F4R forms type-A inert aggregates that do not interact with proteins, but decrease the likelihood of specific enzyme inhibition by decreasing free inhibitor concentration [47]. Thus, type-A inhibitors may result in false negatives in drug screens, leading to bell-shaped dose response curves. In contrast, ESI-09 forms invasive type-B aggregates that non-specifically adsorb proteins and perturb the specific enzyme:substrate interactions, causing false positives in drug screens [47]. In both cases, the key thermodynamic parameters that predict the balance between specific and non-specific binding are the inhibitor’s CAC as well as the K_d_ for the specific interaction. If the CAC is greater than 10* K_d_, the specific enzyme:inhibitor interactions can be investigated, but if the CAC is lower than 10* K_d_, the binding equilibrium shifts toward self-association and aggregation before the enzyme is fully saturated with inhibitor [47]. In the latter case, the inhibitor may not be ideal for biological or pharmacological applications in aqueous systems.

To tailor the use of such inhibitors to more diverse environments, we have also reviewed the mechanism of ABI attenuation by two commonly used agents—TX and HSA. TX remodels invasive type-B co-aggregates into inert type-A assemblies, whereas HSA targets unbound inhibitors [47]. Although useful tools in counter-screens diagnosing ABI, both TX and HSA attenuate non-specific enzyme:inhibitor interactions by competing with specific enzyme:inhibitor interactions [47]. As a result, the attenuators can introduce false negatives in drug screens, discounting potentially viable drug leads. Thus, we advocate the judicious use of such compounds in drug screens after careful consideration of the false positive vs. false negative balance.

Given the ubiquity of ABI and given that most of the currently identified ESIs are low MW, hydrophobic compounds, this review provides mechanistic insight that applies to other ESIs as well. Thus, we hope it to be relevant in modifying existing ESIs, supplementing ESIs with ABI attenuators as well as designing new ESIs with modifications to enhance solubility and decrease aggregation. The elucidation of the mechanisms underlying specific and non-specific interactions of EPAC with its ligands will assist not only the use of these EPAC-modulations as tools to dissect the functional roles of signaling pathways, but also the design of the next generation of EPAC-selective antagonists and agonists.

## Abbreviations

Abbreviation	Description
ABI	aggregation-prone inhibition
BBR	base-binding region
BSA	bovine serum albumin
CAC	critical aggregation concentration
cAMP	cyclic adenosine monophosphate
CBD	cAMP binding domain
CD25HD	classic CD25 homology domain
CHESPA	chemical shift projection analyses
CMC	critical micellar concentration
CNG	cyclic nucleotide gated channels
CR	catalytic region
DLS	dynamic light scattering
EPAC	exchange protein directly activated by cAMP
ESI	EPAC-specific inhibitor
GTP	guanosine triphosphate
GDP	guanosine diphosphate
HCN	hyperpolarization activated cyclic nucleotide gated channels
HSA	human serum albumin
HSQC	heteronuclear single quantum coherence
HTS	high-throughput screen
IL	ionic latch
K_d_	dissociation constant
PBC	phosphate-binding cassette
PKA	cAMP-dependent protein kinase A
Popdc	Popeye domain containing proteins
PRE	paramagnetic relaxation enhancement
RA	Ras association domain
REM	Ras-exchange motif
RR	regulatory region
SLAPSTIC	spin-labels attached to protein side chain
SPR	surface plasmon resonance
STD	saturation transfer difference
TEM	transmission electron microscopy
TX	Triton X100
